# Crystal structures and Hirshfeld surface comparison of fumarate and bromide salts of the etoxazole metabolite designated R7 (aminium ions)

**DOI:** 10.1107/S2056989026002197

**Published:** 2026-03-05

**Authors:** Thaluru M. Mohan Kumar, Chaluvarangaiah Sowbhagya, Papegowda Bhavya, Hemmige S. Yathirajan, Sean Parkin

**Affiliations:** a Department of Physical Sciences Amrita School of Engineering Amrita Vishwa Vidyapeetham, Bengaluru-560 035 India; b Department of Applied Sciences, New Horizon College of Engineering, Bengaluru-560 103, India; c Department of Studies in Chemistry University of Mysore, Manasagangotri Mysuru-570 006 India; d Department of Chemistry University of Kentucky,Lexington KY 40506-0055 USA; Institute of Chemistry, Chinese Academy of Sciences

**Keywords:** crystal structure, Hirshfeld surface, etoxazole metabolite R7, acaricide, insecticide

## Abstract

Etoxazole (C_21_H_23_F_2_NO_2_) is a fluorinated insecticide/acaricide that acts as an inhibitor of chitin biosynthesis. Its degradation products include several persistent environmental contaminants, including a metabolite designated ‘R7’ (C_19_H_25_F_2_NO_3_), which has a readily protonated amine group, to give the R7H^+^ cation, namely, 2-aza­niumyl-2-(4-*tert*-butyl-2-eth­oxy­phen­yl)ethyl 2,6-di­fluoro­benzoate. This paper reports the crystal structures of two salts of R7H^+^: the bromide (**I**: C_21_H_26_F_2_NO_3_^+^·Br^−^) and the 3-carb­oxy­prop-2-enoate (hereafter fumarate) (**II**: C_21_H_26_F_2_NO_3_^+^·C_4_H_3_O_4_^−^). Although the R7H^+^ cations in **I** and **II** are chemically identical, the salts crystallize in substanti­ally different structures. Crystals of **I** are tetra­gonal, space-group type *P*42_1_*c*, whereas those of **II** are monoclinic, space-group type *P*2_1_/*n*, each containing one cation–anion pair in their respective asymmetric units (*Z*′ = 1). The R7H^+^ cation in both structures feature a central 2-aza­niumyl linker connecting 4-*tert*-butyl-2-eth­oxy­phenyl and 2,6-di­fluoro­benzoate rings. Cation conformation is primarily governed by different torsional degrees of freedom, leading to distinct overall conformations. The crystal packing in **I** is driven by N—H⋯Br hydrogen bonds, π–π and C—H⋯π inter­actions, which arrange the cations into columns parallel to the crystallographic *c*-axis. In contrast, the packing in **II** consists of N—H⋯O hydrogen bonds to adjacent fumarate anions, forming extended hydrogen-bonded chains. The fumarate anions are sandwiched between R7H^+^ cations, placing the fumarate double bond between aromatic rings. Hirshfeld-surface analysis shows that inter­molecular contacts in both structures are dominated by inter­actions involving hydrogen (93.4% in **I**, 92.2% in **II**).

## Abstract 2

The crystal structures and Hirshfeld-surface analyses of bromide and fumarate salts of metabolite ’R7′ of the insecticide/acaricide etoxazole are presented.

## 1. Chemical context

Etoxazole [C_21_H_23_F_2_NO_2_, systematic name 4-(4-*tert*-butyl-2-eth­oxy­phen­yl)-2-(2,6-di­fluoro­phen­yl)-4,5-di­hydro-1,3-oxazole], is an oxazoline-based fluorinated insecticide/acaricide, which acts as an inhibitor of chitin biosynthesis. It was developed as a new-generation pesticide to address the growing resistance of spider mites to traditional acaricides, and was selected for development in 1990, achieving registration in 1998 (Suzuki *et al.*, 2002 ▸). This classification reflects its superior bioactivity and novel target compared to established chitin synthesis inhibitors, pyrethroids, and organophosphate (Li *et al.*, 2014 ▸) and has been deployed globally since 1998 (Park *et al.*, 2020 ▸). The synthesis and activity of various 2,4-diphenyl-1,3-oxazolines with acaricidal/insecticidal properties were reported by Suzuki *et al.* (2002 ▸). Metabolic studies of etoxazole by Sun *et al.* (2019 ▸), Macar *et al.* (2022 ▸) and others, have revealed several distinct metabolites resulting from oxidative degradation, especially of its oxazoline ring. These environmental contaminants have been found in plants, earthworms, and soil (Sun *et al.*, 2019 ▸), as well as in rats (Yilmaz *et al.*, 2017 ▸). In view of the agricultural and environmental significance of etoxazole and its metabolites, we have recently reported the crystal structures of etoxazole itself (Sowbhagya *et al.*, 2025*a* ▸), as well as the etoxazole metabolites designated R13 (Mohan Kumar *et al.*, 2024 ▸) and R4 (Sowbhagya *et al.*, 2025*b* ▸). Another related degradation product of etoxazole is the R7 metabolite [C_19_H_25_F_2_NO_3_, systematic name: 2-amino-2-(4-*tert*-butyl-2-eth­oxy­phen­yl)ethyl 2,6-di­fluoro­benzoate], which has an amino group that readily protonates to form an aminium ion, R7H^+^. This paper reports crystal structures of the bromide (**I**: C_21_H_26_BrF_2_NO_3_) and fumarate (**II**: C_25_H_29_F_2_NO_7_) salts of R7H^+^.
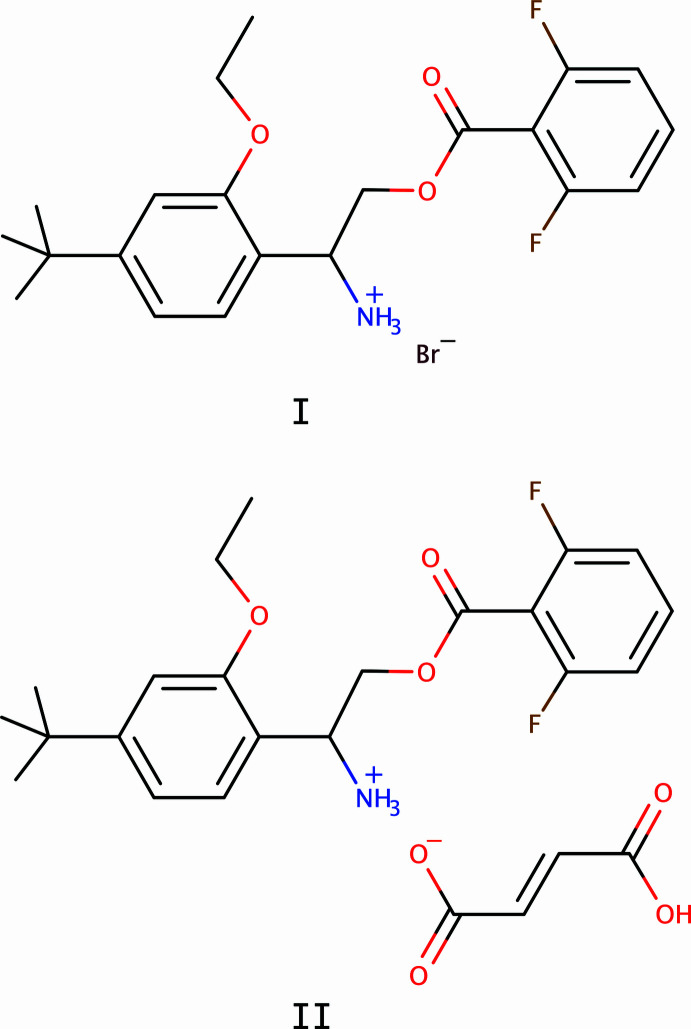


## 2. Structural commentary

In spite of having chemically identical cations, the crystal structures of **I** and **II** are substanti­ally different. Crystals of **I** are tetra­gonal, space-group type *P*
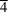
2_1_*c*, whereas those of **II** are monoclinic, space-group type *P*2_1_/*n*, though both have one mol­ecule in their respective asymmetric units (*Z*′ = 1).

**Figure 1:**
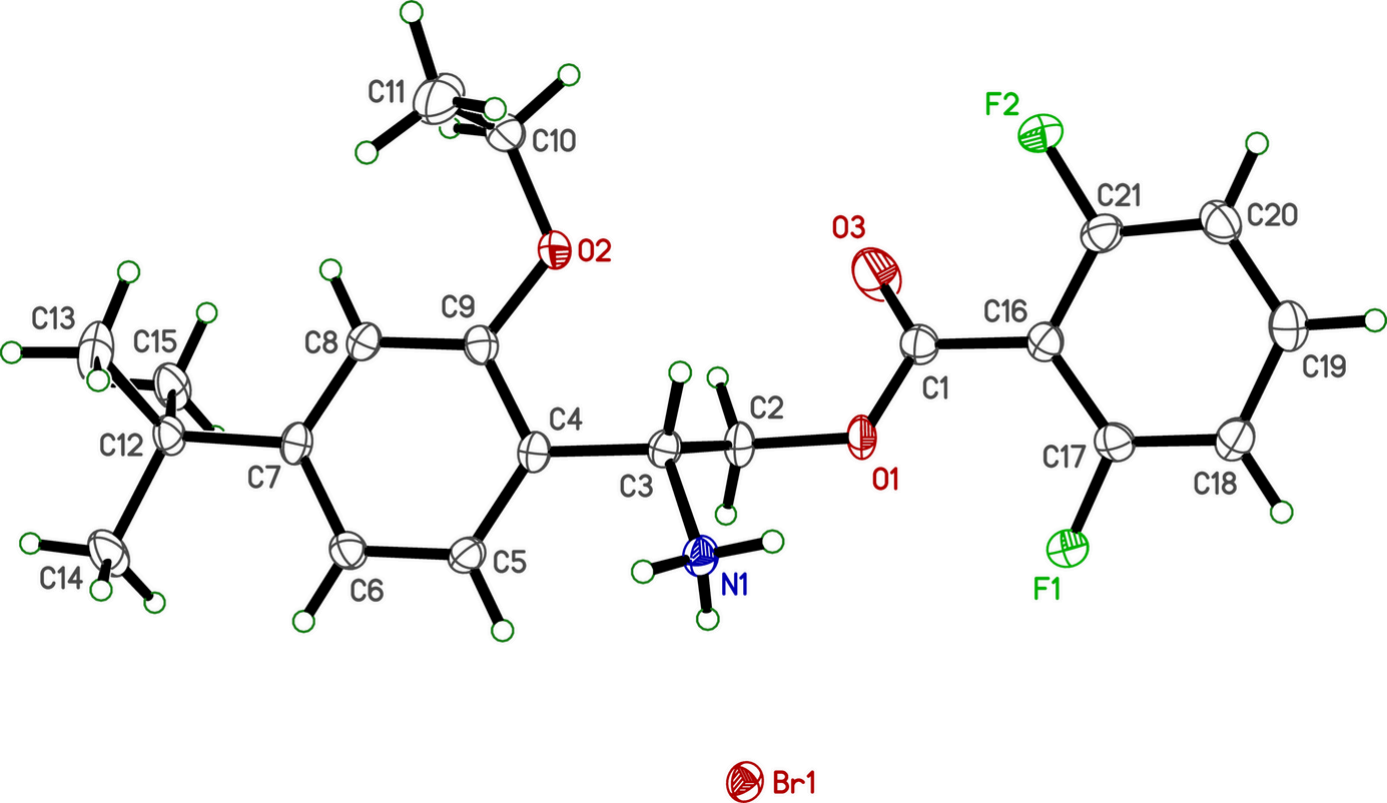
An ellipsoid plot (50% probability) of **I**. Hydrogen atoms are drawn as small arbitrary circles.

**Figure 3:**
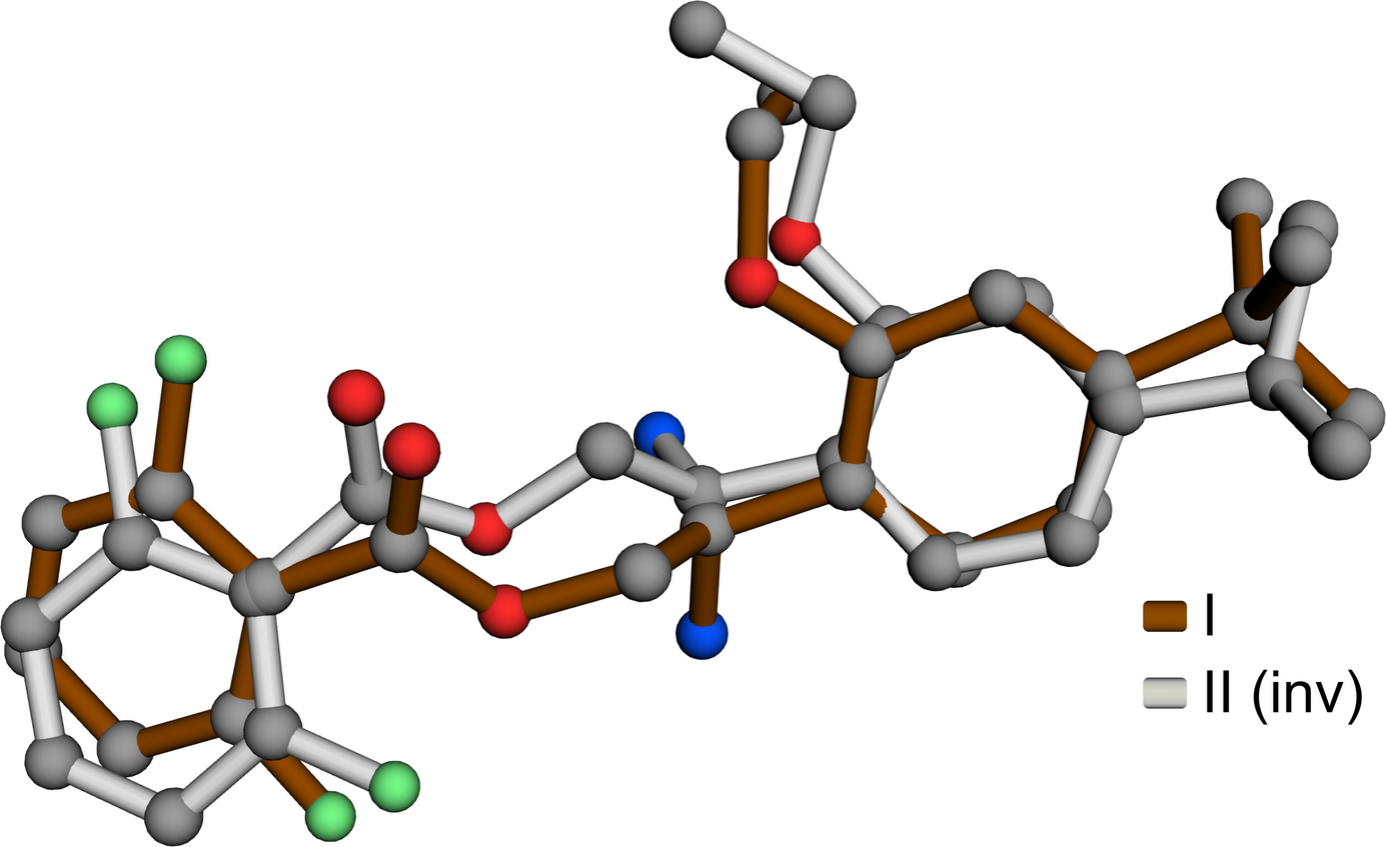
A least-squares-fit overlay of R7H^+^ cations from **I** and **II**.

The R7H^+^ cation in each salt is comprised of a central 2-aza­niumyl (CH_2_CHNH_3_^+^) linker flanked by 4-*tert*-butyl-2-eth­oxy­phenyl and 2,6-di­fluoro­benzoate substituted rings, as shown in Figs. 1 ▸ and 2 ▸. No unusual bond lengths or angles are found in either structure. The conformations of the R7H^+^ cations are governed primarily by torsional degrees of freedom about the C3—C4, C2—C3, O1—C2, and C1—C16 bonds. These are shown qualitatively in a least-squares overlay plot (Fig. 3 ▸) and qu­anti­fied in Table 1 ▸. In the central 2-aza­niumyl moiety, atom C3 is stereogenic , but both structures are strictly racemic (**I** is non-centrosymmetric but possesses a *c*-glide). The chosen asymmetric units for **I** and **II** both had C3 assigned as *R*. A least-squares fit overlay of all 27 non-H cation atoms, however, yields a smaller r.m.s. deviation (1.1328 Å *vs* 1.5544 Å) when one structure is inverted relative to the other. Thus, the *R* enanti­omer of R7H^+^ in **I** has a better overall fit to the *S* enanti­omer of **II** and *vice versa*, which emphasizes the distinct conformations of the two structures.

## 3. Supra­molecular features

**Figure 4:**
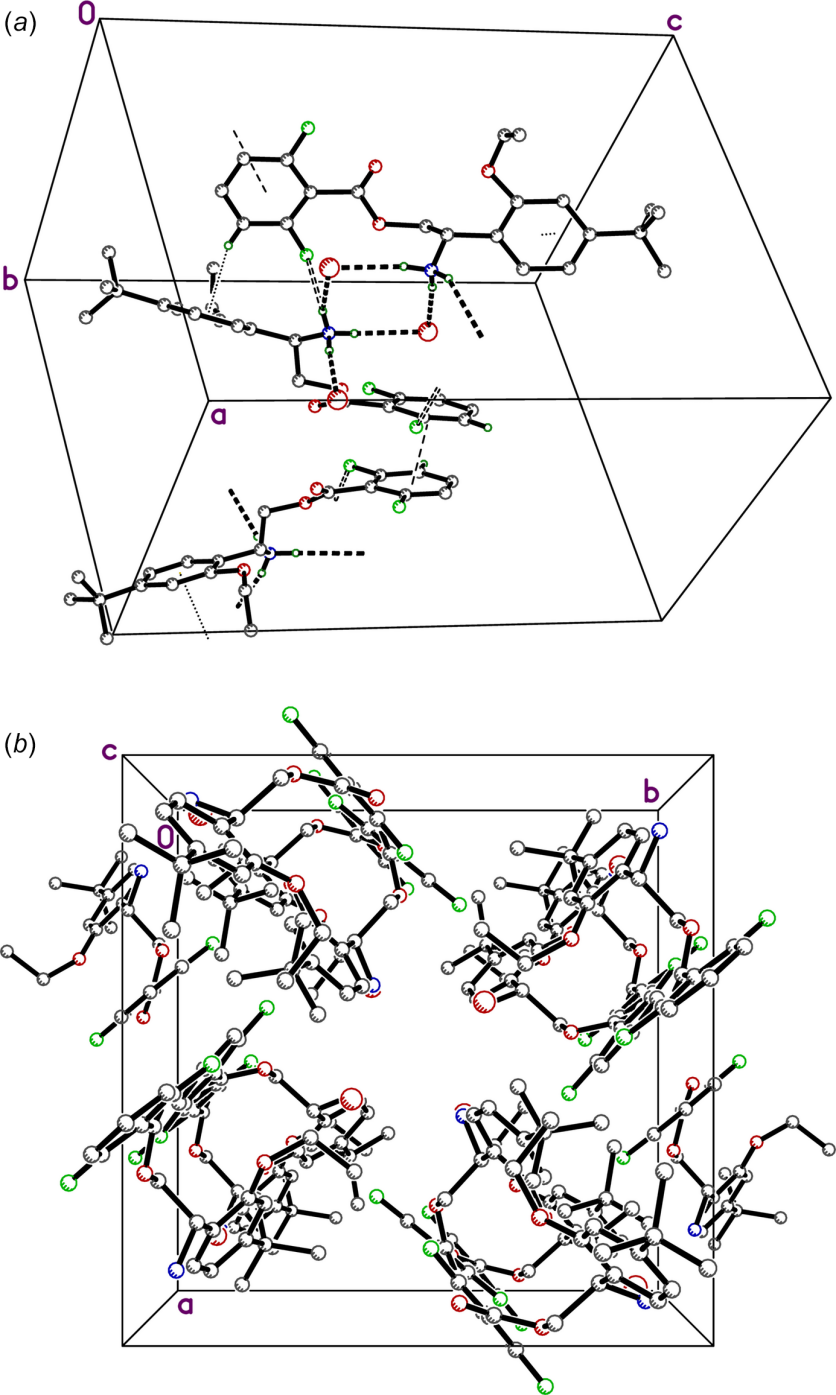
(*a*) A partial packing plot of **I**. Hydrogen bonds are shown as thick dotted lines, N—H⋯F contacts as open dashed lines, π–π stacked rings by thin dashed lines, and C—H⋯π contacts as dotted lines. (*b*) A view down the crystallographic *c*-axis showing the fourfold symmetry.

In **I**, the only conventional hydrogen-bond donor is the ammonium nitro­gen atom, N1, which forms hydrogen bonds to three symmetry-related Br^−^ anions. These are: N1—H1*A*⋯Br1^i^, N1—H1*B*⋯Br1^ii^, and N1—H1C⋯Br1 (symmcodes and distances are given in Table 2 ▸). In addition, the contact N1—H1*A*⋯F1^ii^ and three weak contacts with C—H as donor are flagged as ‘potential hydrogen bonds’ by *SHELXL* (details in Table 2 ▸). There are also π–π and C—H⋯π contacts in **I**. Di­fluoro­benzene ring C16–C21 π-stacks with its counterpart at (1 − *x*, 2 − *y*, *z*) *D_*Cg*⋯*Cg*_* = 3.814 (2) Å, while ring C4–C9 is in close contact with H18^ii^, *D_*Cg*_*_⋯H_ = 2.73 Å. Each type of inter­action is shown in Fig. 4 ▸*a.* In combination, these contacts connect the R7H^+^ cations into columns parallel to the *c*-axis, which pack in a square array to give the overall tetra­gonal packing symmetry, as shown in Fig. 4 ▸*b*.

**Figure 5:**
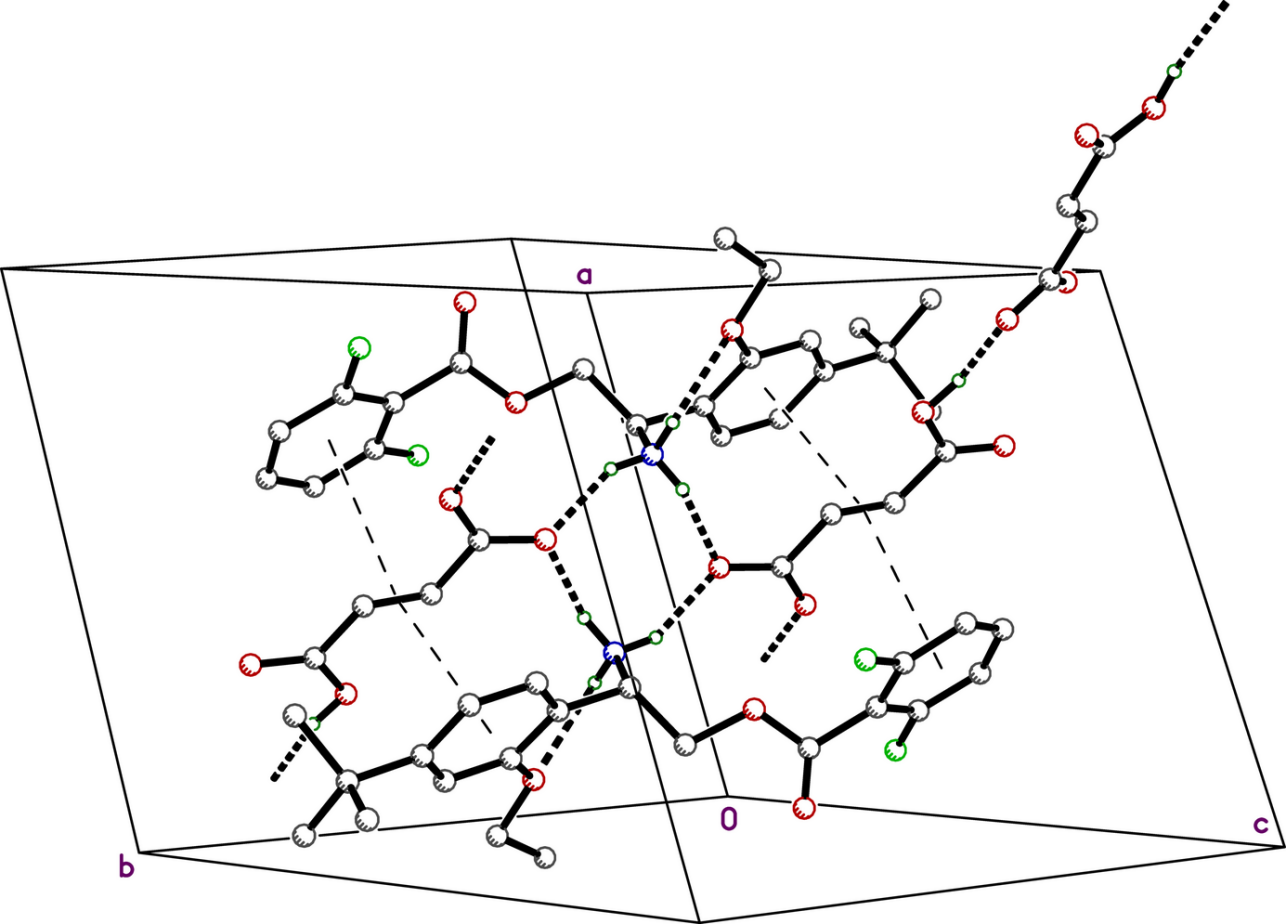
A partial packing plot of **II**. Hydrogen bonds are shown as thick dotted lines and overlap of the fumarate double bond with aromatic rings as thin dashed lines.

In **II**, the R7H^+^ ammonium group also forms three conventional hydrogen bonds. Two of these are to O4 of adjacent fumarate anions, namely N1—H1*C*⋯O4 and N1—H1*A*⋯O4^i^ (distances, angles and symmcodes are given in Table 3 ▸). The remaining hydrogen bond is intra­molecular, N1—H1*B*⋯O2, within the R7H^+^ cation. Atom H1*B* is also in close contact with a symmetry-related fumarate O7, *i.e*., N1—H1*B*⋯O7^ii^, but the angle is small (111.5°, Table 3 ▸). In addition, the fumarate anions form extended O6—H6*A*⋯O5^iv^ hydrogen-bonded chains. As in **I**, there are also weaker contacts with C—H as donor, which are also given in Table 3 ▸. There is no π–π stacking of rings in **I**, but the central double bond of the fumarate anion sits sandwiched between the planes of ring C4–C9 and C16–C19^i^ at a distances of 3.512 and 3.636, Å, respectively between ring and double-bond centroids. These inter­actions are shown in Fig. 5 ▸.

**Figure 6:**
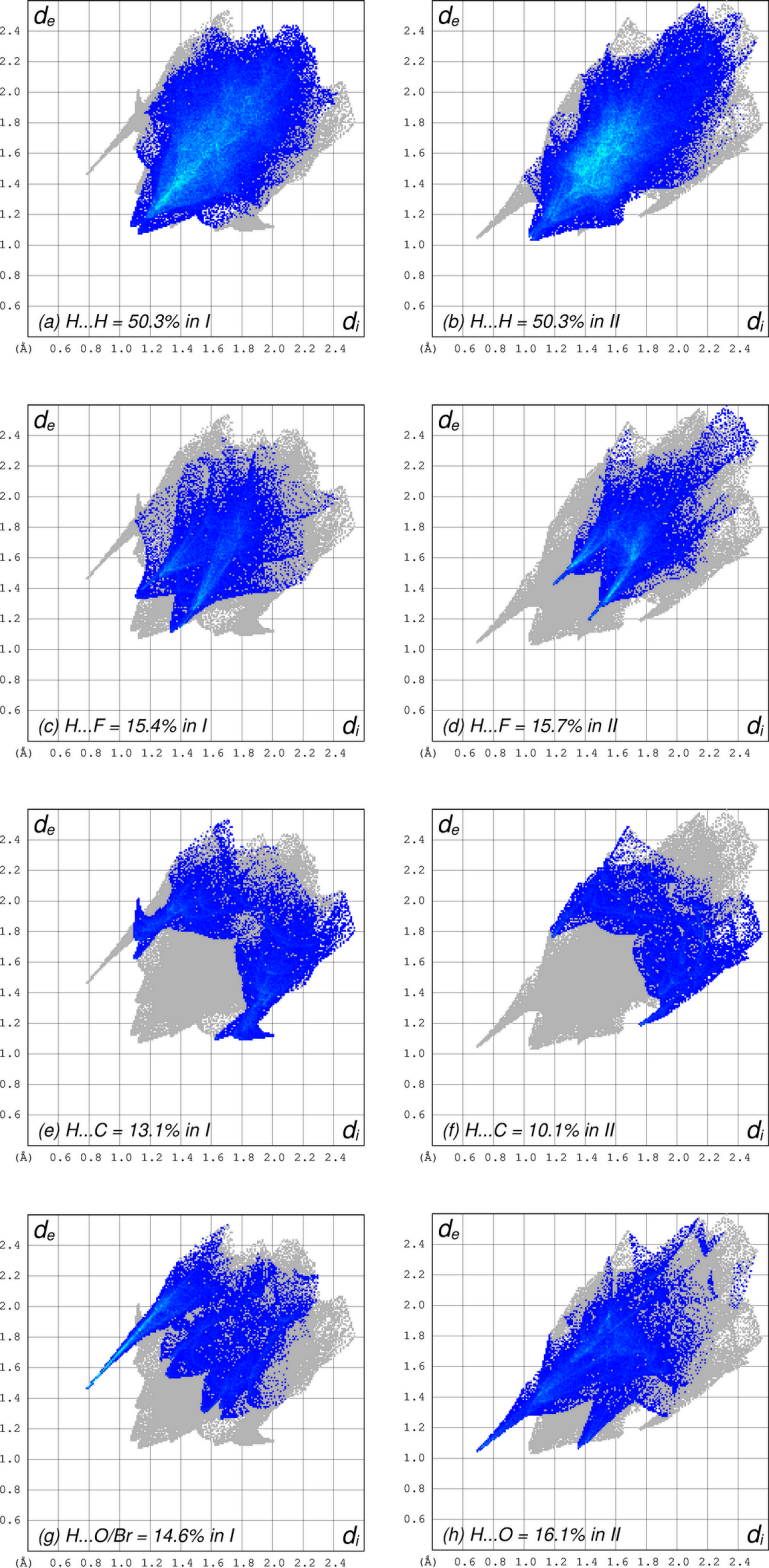
Hirshfeld-surface fingerprint plots of the most abundant types of atom-atom contacts in **I** and **II**. (*a*),(*b*) H⋯H contacts, (*c*),(*d*) H⋯F, (*e*),(*f*) H⋯C, (*g*),(*h*) H⋯O/Br and H⋯O contacts. For ease of comparison of the hydrogen-bonding contacts of the ammonium cation, panel (*g*) is a composite of H⋯O and H⋯Br contacts.

A Hirshfeld surface analysis using *CrystalExplorer* (Spackman *et al.*, 2021 ▸) shows that the most important inter­molecular contacts for the R7H^+^ cation in **I** and **II** involve hydrogen (93.4% in **I**, 92.2% in **II**), with over 50% being H⋯H in both structures. These are summarized pairwise for ease of comparison in Fig. 6 ▸.

## 4. Database survey

A search of the Cambridge Structural Database (CSD v6.0, April 2025: Groom *et al.*, 2016 ▸) using a search fragment consisting of the R7 backbone but with the *tert*-butyl, eth­oxy, and fluoride substituents set to ‘any group’ and the carbon–nitro­gen and carbon­yl–group bonds set to ‘any type’ returned 64 hits. Of these, none includes either unsubstituted amino (–NH_2_) or ammonium (–NH_3_^+^) groups. Furthermore, only three structures include fluorine substituents: CSD refcodes AFABUE (Maligres *et al.*, 2002 ▸) and CIBKUT (Seiler *et al.*, 1999 ▸) have –CF_3_ groups, while LEZRIU (Xing *et al.*, 2018 ▸) has a single fluorine atom at the 4-position of the benzoate ring, but otherwise have little in common with R7. Other related structures include the parent mol­ecule etoxazole (DULGUQ: Sowbhagya *et al.*, 2025*a* ▸) and its R4 (ULOVAW: Sowbhagya *et al.*, 2025*b* ▸) and R13 (UGUQUM: Mohan Kumar *et al.*, 2024 ▸) metabolites.

## 5. Synthesis and crystallization

Solutions of the etoxazole metabolite R7 (100 mg, 0.265 mmol) in methanol (5 ml) were mixed with either equimolar qu­anti­ties of a methano­lic solution of fumaric acid (31 mg) or 2 ml of HBr (48% aqueous solution) in 10 ml of methanol and stirred for an hour at 313 K. The mixtures were set aside for slow evaporation for 24 h. The resulting crystals, suitable for X-ray diffraction analysis, were collected by filtration and air dried.

## 6. Refinement

Crystal data, data collection and structure refinement details are summarized in Table 4 ▸. All hydrogen atoms were found in difference-Fourier maps, but subsequently included in the refinement using riding models. Carbon-bound hydrogen atoms were affixed with constrained distances set to 0.95 Å (C*sp*^2^—H), 0.98 Å (*R*CH_3_), 0.99 Å (*R*_2_CH_2_) and 1.00 Å (*R*_3_CH). Hydrogen atoms bound to nitro­gen and oxygen used riding models that allowed their bond distances to refine. *U*_iso_(H) parameters were set to values of either 1.2*U*_eq_ or 1.5*U*_eq_ (*R*CH_3_, *R*NH_3_, OH) of the attached atom.

## Supplementary Material

Crystal structure: contains datablock(s) I, II, global. DOI: 10.1107/S2056989026002197/nx2032sup1.cif

Structure factors: contains datablock(s) I. DOI: 10.1107/S2056989026002197/nx2032Isup2.hkl

Structure factors: contains datablock(s) II. DOI: 10.1107/S2056989026002197/nx2032IIsup3.hkl

Supporting information file. DOI: 10.1107/S2056989026002197/nx2032Isup4.cml

Supporting information file. DOI: 10.1107/S2056989026002197/nx2032IIsup5.cml

CCDC references: 2533698, 2533697

Additional supporting information:  crystallographic information; 3D view; checkCIF report

## Figures and Tables

**Figure 2:**
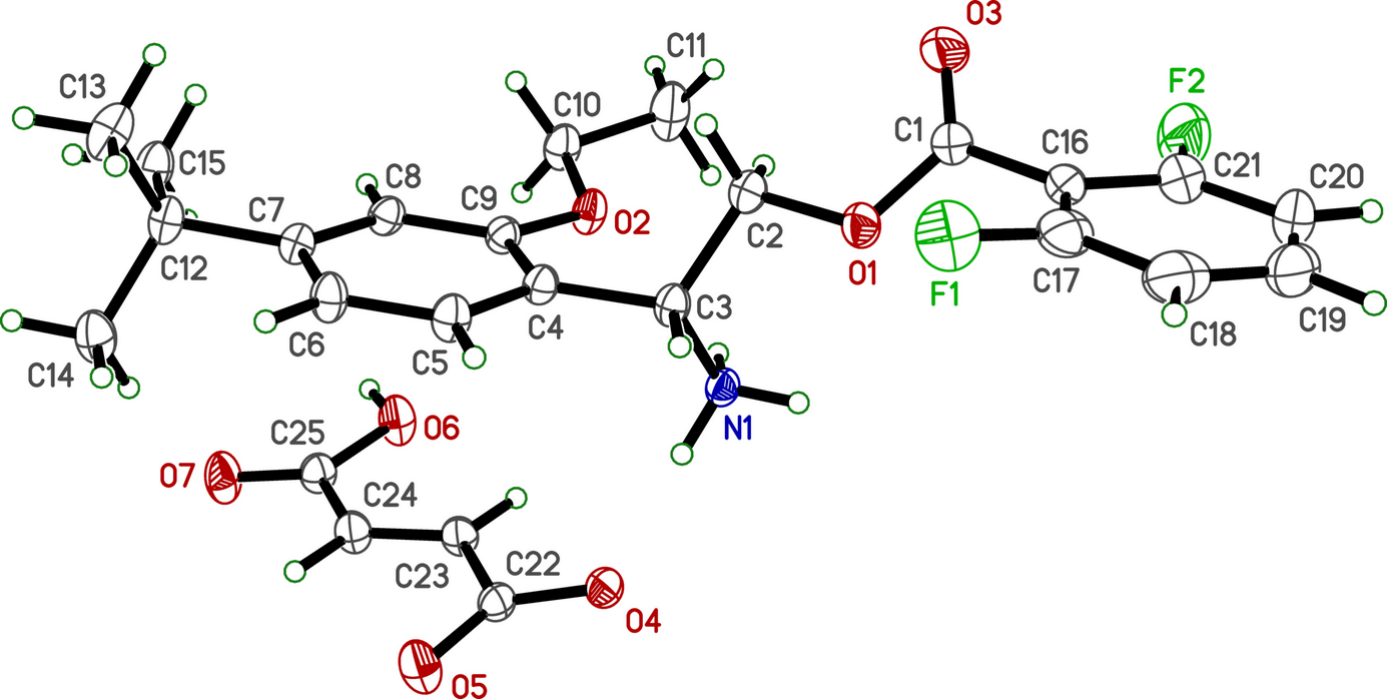
An ellipsoid plot (50% probability) of **II**. Hydrogen atoms are drawn as small arbitrary circles.

**Table 1:** Conformation-defining torsion angles (°) in **I** and **II**

Torsion angle	**I**	**II**
C5—C4—C3—C2	98.7 (3)	−117.45 (12)
C4—C3—C2—O1	178.0 (2)	160.96 (9)
C3—C2—O1—C1	−120.5 (3)	170.25 (9)
C2—O1—C1—C16	175.2 (2)	175.71 (10)
O1—C1—C16—C21	−144.0 (3)	134.09 (12)
N1—C3—C2—O1	−58.2 (3)	−73.64 (10)
N1—C3—C4—C9	155.1 (3)	−61.61 (13)
C9—O2—C10—C11	67.6 (4)	164.55 (11)
C4—C9—O2—C10	−161.5 (3)	−168.79 (10)
C8—C7—C13—C14	165.0 (2)	123.99 (12)

**Table 2:** Hydrogen-bond geometry (Å, °) for **I**[Chem scheme1]

*D*—H⋯*A*	*D*—H	H⋯*A*	*D*⋯*A*	*D*—H⋯*A*
N1—H1*A*⋯Br1^i^	0.88	2.57	3.337 (2)	147
N1—H1*A*⋯F1^ii^	0.88	2.52	3.073 (3)	122
N1—H1*B*⋯Br1^ii^	0.88	2.39	3.259 (2)	170
N1—H1*C*⋯Br1	0.88	2.38	3.252 (2)	175
C10—H10*A*⋯Br1^iii^	0.99	2.75	3.650 (3)	152
C2—H2*B*⋯O2	0.99	2.62	3.116 (4)	111
C8—H8⋯Br1^iii^	0.95	3.10	4.040 (3)	170

Symmetry codes: (i) 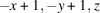; (ii) 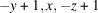; (iii) 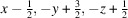.

**Table 3:** Hydrogen-bond geometry (Å, °) for **II**[Chem scheme1]

*D*—H⋯*A*	*D*—H	H⋯*A*	*D*⋯*A*	*D*—H⋯*A*
N1—H1*A*⋯O4^i^	0.89	1.89	2.7160 (12)	152
N1—H1*B*⋯O2	0.89	2.30	2.8889 (12)	123
N1—H1*B*⋯O7^ii^	0.89	2.36	2.8103 (13)	112
N1—H1*C*⋯O4	0.89	1.84	2.6915 (12)	158
C2—H2*A*⋯O7^ii^	0.99	2.53	3.1832 (15)	123
C2—H2*B*⋯O3^iii^	0.99	2.48	3.1727 (15)	127
O6—H6*A*⋯O5^iv^	0.92	1.62	2.5184 (11)	165

Symmetry codes: (i) 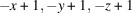; (ii) 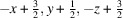; (iii) 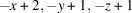; (iv) 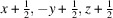.

**Table 4:** Experimental details

	**I**	**II**
Crystal data		
Chemical formula	C_21_H_26_F_2_NO_3_^+^·Br^−^	C_21_H_26_F_2_NO_3_^+^·C_4_H_3_O_4_^−^
*M*_r_	458.34	493.49
Crystal system, space group	Tetragonal, *P* 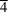 2_1_*c*	Monoclinic, *P*2_1_/*n*
Temperature (K)	100	100
*a*, *b*, *c* (Å)	15.2984 (2), 15.2984 (2), 17.8818 (3)	12.0615 (2), 15.3673 (3), 14.0141 (3)
α, β, γ (°)	90, 90, 90	90, 110.506 (1), 90
*V* (Å^3^)	4185.07 (13)	2432.96 (8)
*Z*	8	4
Radiation type	Mo *K*α	Mo *K*α
μ (mm^−1^)	2.00	0.11
Crystal size (mm)	0.21 × 0.19 × 0.18	0.19 × 0.18 × 0.12

Data collection		
Diffractometer	Bruker D8 Venture dual source	Bruker D8 Venture dual source
Absorption correction	Multi-scan (*SADABS*; Krause *et al.*, 2015 ▸)	Multi-scan (*SADABS*; Krause *et al.*, 2015 ▸)
*T*_min_, *T*_max_	0.830, 0.928	0.917, 0.971
No. of measured, independent and observed [*I* > 2σ(*I*)] reflections	75181, 4795, 4496	42339, 5576, 4976
*R*_int_	0.049	0.031
(sin θ/λ)_max_ (Å^−1^)	0.650	0.650

Refinement		
*R*[*F*^2^ > 2σ(*F*^2^)], *wR*(*F*^2^), *S*	0.024, 0.058, 1.03	0.036, 0.101, 1.03
No. of reflections	4795	5576
No. of parameters	259	324
H-atom treatment	H atoms treated by a mixture of independent and constrained refinement	H atoms treated by a mixture of independent and constrained refinement
Δρ_max_, Δρ_min_ (e Å^−3^)	0.80, −0.44	0.34, −0.21
Absolute structure	Flack *x* determined using 1911 quotients [(*I*^+^)−(*I*^−^)]/[(*I*^+^)+(*I*^−^)] (Parsons *et al.*, 2013 ▸)	–
Absolute structure parameter	0.018 (3)	–

Computer programs: *APEX5* (Bruker, 2023 ▸), *SHELXT* (Sheldrick, 2015*a* ▸), *SHELXL2025/1* (Sheldrick, 2015*b* ▸), *XP in *SHELXTL** (Sheldrick, 2008 ▸), *over-lay* (Parkin, 2025 ▸) and *publCIF* (Westrip, 2010 ▸).

## supplementary crystallographic information

### 2-Azaniumyl-2-(4-tert-butyl-2-ethoxyphenyl)ethyl 2,6-difluorobenzoate bromide (I) Crystal data

**Table:**

C_21_H_26_F_2_NO_3_^+^·Br^−^	*D*_x_ = 1.455 Mg m^−^^3^
*M**_r_* = 458.34	Mo *K*α radiation, λ = 0.71073 Å
Tetragonal, *P*42_1_*c*	Cell parameters from 9610 reflections
*a* = 15.2984 (2) Å	θ = 2.6–27.5°
*c* = 17.8818 (3) Å	µ = 2.00 mm^−^^1^
*V* = 4185.07 (13) Å^3^	*T* = 100 K
*Z* = 8	Irregular block, colourless
*F*(000) = 1888	0.21 × 0.19 × 0.18 mm

### 2-Azaniumyl-2-(4-tert-butyl-2-ethoxyphenyl)ethyl 2,6-difluorobenzoate bromide (I) Data collection

**Table:**

Bruker D8 Venture dual source diffractometer	4795 independent reflections
Radiation source: microsource	4496 reflections with *I* > 2σ(*I*)
Detector resolution: 7.41 pixels mm^-1^	*R*_int_ = 0.049
φ and ω scans	θ_max_ = 27.5°, θ_min_ = 1.9°
Absorption correction: multi-scan (*SADABS*; Krause *et al*., 2015)	*h* = −19→19
*T*_min_ = 0.830, *T*_max_ = 0.928	*k* = −19→16
75181 measured reflections	*l* = −23→23

### 2-Azaniumyl-2-(4-tert-butyl-2-ethoxyphenyl)ethyl 2,6-difluorobenzoate bromide (I) Refinement

**Table:**

Refinement on *F*^2^	Secondary atom site location: difference Fourier map
Least-squares matrix: full	Hydrogen site location: difference Fourier map
*R*[*F*^2^ > 2σ(*F*^2^)] = 0.024	H atoms treated by a mixture of independent and constrained refinement
*wR*(*F*^2^) = 0.058	*w* = 1/[σ^2^(*F*_o_^2^) + (0.0243*P*)^2^ + 2.4223*P*] where *P* = (*F*_o_^2^ + 2*F*_c_^2^)/3
*S* = 1.03	(Δ/σ)_max_ = 0.002
4795 reflections	Δρ_max_ = 0.80 e Å^−^^3^
259 parameters	Δρ_min_ = −0.44 e Å^−^^3^
0 restraints	Absolute structure: Flack *x* determined using 1911 quotients [(*I*^+^)-(*I*^-^)]/[(*I*^+^)+(*I*^-^)] (Parsons *et al.*, 2013)
Primary atom site location: structure-invariant direct methods	Absolute structure parameter: 0.018 (3)

### 2-Azaniumyl-2-(4-tert-butyl-2-ethoxyphenyl)ethyl 2,6-difluorobenzoate bromide (I) Special details

**Table:**

Experimental. The crystal was mounted using polyisobutene oil on the tip of a fine glass fibre, which was fastened in a copper mounting pin with electrical solder. It was placed directly into the cold gas stream of a liquid-nitrogen based cryostat (Hope, 1994; Parkin & Hope, 1998). Diffraction data were collected with the crystal at 100K.
Geometry. All esds (except the esd in the dihedral angle between two l.s. planes) are estimated using the full covariance matrix. The cell esds are taken into account individually in the estimation of esds in distances, angles and torsion angles; correlations between esds in cell parameters are only used when they are defined by crystal symmetry. An approximate (isotropic) treatment of cell esds is used for estimating esds involving l.s. planes.
Refinement. Refinement progress was checked using *Platon* (Spek, 2020) and by an *R*-tensor (Parkin, 2000). The final model was further checked with the IUCr utility *checkCIF*.

### 2-Azaniumyl-2-(4-tert-butyl-2-ethoxyphenyl)ethyl 2,6-difluorobenzoate bromide (I) Fractional atomic coordinates and isotropic or equivalent isotropic displacement parameters (Å^2^)

**Table:**

	*x*	*y*	*z*	*U*_iso_*/*U*_eq_	
Br1	0.62255 (2)	0.58962 (2)	0.39254 (2)	0.01946 (8)	
F1	0.57938 (12)	0.78020 (12)	0.58387 (9)	0.0244 (4)	
F2	0.34194 (13)	0.97753 (13)	0.58768 (10)	0.0315 (5)	
O1	0.46509 (15)	0.79073 (13)	0.47217 (11)	0.0210 (5)	
O2	0.27044 (14)	0.80263 (14)	0.31527 (11)	0.0218 (4)	
O3	0.4237 (2)	0.93037 (16)	0.45861 (13)	0.0469 (8)	
N1	0.41590 (16)	0.62168 (16)	0.42530 (12)	0.0179 (4)	
H1A	0.3844 (11)	0.5764 (10)	0.4118 (9)	0.027*	
H1B	0.4117 (13)	0.6290 (6)	0.4739 (10)	0.027*	
H1C	0.4708 (12)	0.6127 (8)	0.4132 (9)	0.027*	
C1	0.4477 (2)	0.8717 (2)	0.49735 (16)	0.0212 (6)	
C2	0.45015 (19)	0.77420 (19)	0.39362 (18)	0.0209 (6)	
H2A	0.505536	0.756944	0.369078	0.025*	
H2B	0.428066	0.827752	0.368948	0.025*	
C3	0.38341 (18)	0.70115 (18)	0.38652 (15)	0.0173 (5)	
H3	0.328393	0.720397	0.411841	0.021*	
C4	0.36260 (19)	0.68309 (19)	0.30538 (15)	0.0170 (6)	
C5	0.39957 (18)	0.61581 (19)	0.26437 (14)	0.0188 (6)	
H5	0.441988	0.579018	0.287063	0.023*	
C6	0.3753 (2)	0.60129 (19)	0.19004 (15)	0.0201 (6)	
H6	0.401034	0.554458	0.162943	0.024*	
C7	0.31381 (19)	0.65487 (19)	0.15526 (15)	0.0170 (6)	
C8	0.27756 (19)	0.7236 (2)	0.19644 (16)	0.0175 (6)	
H8	0.236174	0.761410	0.173554	0.021*	
C9	0.30155 (19)	0.73723 (19)	0.27069 (15)	0.0171 (6)	
C10	0.1902 (2)	0.8458 (2)	0.29526 (18)	0.0290 (7)	
H10A	0.194533	0.867321	0.243186	0.035*	
H10B	0.181494	0.896979	0.328314	0.035*	
C11	0.1127 (2)	0.7860 (3)	0.30182 (19)	0.0391 (9)	
H11A	0.058984	0.819086	0.292220	0.059*	
H11B	0.110555	0.761248	0.352339	0.059*	
H11C	0.117919	0.738644	0.265185	0.059*	
C12	0.2838 (2)	0.64002 (19)	0.07426 (15)	0.0195 (6)	
C13	0.1847 (2)	0.6239 (3)	0.07394 (19)	0.0356 (8)	
H13A	0.165704	0.609215	0.023144	0.053*	
H13B	0.154416	0.676820	0.090753	0.053*	
H13C	0.170823	0.575431	0.107730	0.053*	
C14	0.3291 (3)	0.5618 (2)	0.03807 (17)	0.0317 (8)	
H14A	0.309098	0.555738	−0.013747	0.048*	
H14B	0.314701	0.508575	0.065992	0.048*	
H14C	0.392483	0.570840	0.038724	0.048*	
C15	0.3045 (2)	0.7211 (2)	0.02736 (17)	0.0266 (7)	
H15A	0.284634	0.711835	−0.024144	0.040*	
H15B	0.367679	0.731538	0.027601	0.040*	
H15C	0.274350	0.771924	0.048583	0.040*	
C16	0.45887 (19)	0.87692 (19)	0.58045 (16)	0.0184 (6)	
C17	0.52045 (19)	0.82971 (19)	0.62108 (16)	0.0197 (6)	
C18	0.5267 (2)	0.8323 (2)	0.69799 (16)	0.0220 (6)	
H18	0.570311	0.799695	0.723517	0.026*	
C19	0.4672 (2)	0.8839 (2)	0.73735 (17)	0.0244 (7)	
H19	0.469349	0.885533	0.790432	0.029*	
C20	0.4056 (2)	0.9324 (2)	0.69972 (17)	0.0234 (6)	
H20	0.365103	0.967676	0.726479	0.028*	
C21	0.4032 (2)	0.92928 (19)	0.62289 (17)	0.0220 (6)	

### 2-Azaniumyl-2-(4-tert-butyl-2-ethoxyphenyl)ethyl 2,6-difluorobenzoate bromide (I) Atomic displacement parameters (Å^2^)

**Table:**

	*U*^11^	*U*^22^	*U*^33^	*U*^12^	*U*^13^	*U*^23^
Br1	0.01987 (14)	0.02421 (15)	0.01431 (11)	0.00078 (11)	0.00101 (12)	0.00146 (12)
F1	0.0237 (9)	0.0264 (9)	0.0230 (9)	0.0065 (8)	−0.0018 (7)	−0.0009 (7)
F2	0.0326 (10)	0.0308 (10)	0.0312 (11)	0.0145 (8)	−0.0093 (8)	−0.0051 (8)
O1	0.0334 (12)	0.0155 (10)	0.0140 (9)	−0.0006 (9)	−0.0079 (8)	−0.0011 (8)
O2	0.0258 (11)	0.0241 (11)	0.0154 (9)	0.0063 (9)	−0.0050 (8)	−0.0046 (8)
O3	0.089 (2)	0.0268 (13)	0.0252 (11)	0.0228 (14)	−0.0070 (14)	0.003 (1)
N1	0.0205 (12)	0.0193 (12)	0.014 (1)	−0.0003 (12)	−0.0012 (9)	0.0008 (9)
C1	0.0248 (15)	0.0183 (14)	0.0205 (14)	0.0000 (13)	−0.0014 (11)	0.0000 (12)
C2	0.0263 (15)	0.0237 (14)	0.0127 (11)	−0.0035 (11)	−0.0033 (13)	−0.0004 (13)
C3	0.0195 (13)	0.0193 (13)	0.0130 (12)	0.0013 (11)	−0.0013 (12)	0.0018 (11)
C4	0.0188 (14)	0.0188 (14)	0.0135 (13)	−0.0028 (11)	−0.0015 (10)	0.0009 (10)
C5	0.0182 (14)	0.0214 (15)	0.0170 (12)	0.0033 (12)	−0.0017 (10)	0.0013 (11)
C6	0.0246 (15)	0.0191 (15)	0.0167 (12)	0.0030 (13)	−0.0005 (12)	−0.0009 (10)
C7	0.0185 (14)	0.0199 (14)	0.0127 (12)	−0.0046 (12)	−0.0014 (11)	0.0027 (11)
C8	0.0178 (14)	0.0185 (14)	0.0161 (13)	−0.0009 (11)	−0.0023 (11)	0.0020 (11)
C9	0.0179 (14)	0.0190 (14)	0.0145 (13)	−0.0020 (11)	0.0014 (11)	−0.0002 (11)
C10	0.0338 (18)	0.0326 (18)	0.0206 (15)	0.0144 (15)	−0.0054 (13)	−0.0057 (13)
C11	0.0228 (18)	0.067 (3)	0.0278 (18)	0.0075 (18)	0.0006 (14)	−0.0051 (17)
C12	0.0276 (16)	0.0190 (15)	0.0118 (12)	0.0013 (12)	−0.0031 (11)	−0.0007 (11)
C13	0.0316 (18)	0.053 (2)	0.0224 (15)	−0.0098 (18)	−0.0063 (14)	−0.0077 (16)
C14	0.054 (2)	0.0239 (17)	0.0171 (14)	0.0089 (16)	−0.0062 (14)	−0.0036 (12)
C15	0.041 (2)	0.0237 (16)	0.0150 (14)	−0.0003 (15)	−0.0021 (13)	0.0007 (12)
C16	0.0206 (14)	0.0142 (13)	0.0203 (13)	−0.0025 (12)	−0.0029 (11)	−0.0005 (11)
C17	0.0210 (14)	0.0147 (14)	0.0235 (15)	−0.0017 (11)	−0.0008 (11)	−0.0017 (11)
C18	0.0227 (16)	0.0217 (16)	0.0216 (15)	−0.0008 (13)	−0.0044 (12)	0.0011 (12)
C19	0.0284 (16)	0.0243 (16)	0.0205 (14)	−0.0049 (13)	−0.0023 (12)	−0.0018 (13)
C20	0.0237 (15)	0.0227 (16)	0.0239 (14)	−0.0010 (13)	0.0008 (12)	−0.0065 (12)
C21	0.0208 (14)	0.0180 (14)	0.0273 (16)	0.0008 (11)	−0.0050 (11)	−0.0009 (11)

### 2-Azaniumyl-2-(4-tert-butyl-2-ethoxyphenyl)ethyl 2,6-difluorobenzoate bromide (I) Geometric parameters (Å, º)

**Table:**

F1—C17	1.353 (3)	C10—C11	1.502 (5)
F2—C21	1.349 (3)	C10—H10A	0.9900
O1—C1	1.345 (4)	C10—H10B	0.9900
O1—C2	1.445 (4)	C11—H11A	0.9800
O2—C9	1.365 (3)	C11—H11B	0.9800
O2—C10	1.439 (4)	C11—H11C	0.9800
O3—C1	1.192 (4)	C12—C14	1.527 (4)
N1—C3	1.485 (3)	C12—C15	1.531 (4)
N1—H1A	0.878 (18)	C12—C13	1.535 (5)
N1—H1B	0.878 (18)	C13—H13A	0.9800
N1—H1C	0.878 (18)	C13—H13B	0.9800
C1—C16	1.498 (4)	C13—H13C	0.9800
C2—C3	1.519 (4)	C14—H14A	0.9800
C2—H2A	0.9900	C14—H14B	0.9800
C2—H2B	0.9900	C14—H14C	0.9800
C3—C4	1.511 (4)	C15—H15A	0.9800
C3—H3	1.0000	C15—H15B	0.9800
C4—C5	1.385 (4)	C15—H15C	0.9800
C4—C9	1.394 (4)	C16—C17	1.392 (4)
C5—C6	1.398 (4)	C16—C21	1.393 (4)
C5—H5	0.9500	C17—C18	1.379 (4)
C6—C7	1.395 (4)	C18—C19	1.395 (4)
C6—H6	0.9500	C18—H18	0.9500
C7—C8	1.399 (4)	C19—C20	1.377 (5)
C7—C12	1.536 (4)	C19—H19	0.9500
C8—C9	1.393 (4)	C20—C21	1.375 (4)
C8—H8	0.9500	C20—H20	0.9500
			
C1—O1—C2	117.1 (2)	C10—C11—H11B	109.5
C9—O2—C10	119.2 (2)	H11A—C11—H11B	109.5
C3—N1—H1A	109.5	C10—C11—H11C	109.5
C3—N1—H1B	109.5	H11A—C11—H11C	109.5
H1A—N1—H1B	109.5	H11B—C11—H11C	109.5
C3—N1—H1C	109.5	C14—C12—C15	108.0 (3)
H1A—N1—H1C	109.5	C14—C12—C13	108.7 (3)
H1B—N1—H1C	109.5	C15—C12—C13	109.4 (3)
O3—C1—O1	124.1 (3)	C14—C12—C7	112.3 (3)
O3—C1—C16	124.9 (3)	C15—C12—C7	109.6 (2)
O1—C1—C16	111.0 (3)	C13—C12—C7	108.8 (2)
O1—C2—C3	108.4 (2)	C12—C13—H13A	109.5
O1—C2—H2A	110.0	C12—C13—H13B	109.5
C3—C2—H2A	110.0	H13A—C13—H13B	109.5
O1—C2—H2B	110.0	C12—C13—H13C	109.5
C3—C2—H2B	110.0	H13A—C13—H13C	109.5
H2A—C2—H2B	108.4	H13B—C13—H13C	109.5
N1—C3—C4	111.7 (2)	C12—C14—H14A	109.5
N1—C3—C2	109.8 (2)	C12—C14—H14B	109.5
C4—C3—C2	110.9 (2)	H14A—C14—H14B	109.5
N1—C3—H3	108.1	C12—C14—H14C	109.5
C4—C3—H3	108.1	H14A—C14—H14C	109.5
C2—C3—H3	108.1	H14B—C14—H14C	109.5
C5—C4—C9	118.7 (2)	C12—C15—H15A	109.5
C5—C4—C3	124.0 (3)	C12—C15—H15B	109.5
C9—C4—C3	117.4 (3)	H15A—C15—H15B	109.5
C4—C5—C6	120.9 (3)	C12—C15—H15C	109.5
C4—C5—H5	119.6	H15A—C15—H15C	109.5
C6—C5—H5	119.6	H15B—C15—H15C	109.5
C7—C6—C5	120.6 (3)	C17—C16—C21	115.3 (3)
C7—C6—H6	119.7	C17—C16—C1	124.6 (3)
C5—C6—H6	119.7	C21—C16—C1	120.1 (3)
C6—C7—C8	118.4 (3)	F1—C17—C18	117.4 (3)
C6—C7—C12	122.4 (3)	F1—C17—C16	119.0 (3)
C8—C7—C12	119.3 (3)	C18—C17—C16	123.5 (3)
C9—C8—C7	120.6 (3)	C17—C18—C19	118.3 (3)
C9—C8—H8	119.7	C17—C18—H18	120.8
C7—C8—H8	119.7	C19—C18—H18	120.8
O2—C9—C8	125.1 (3)	C20—C19—C18	120.4 (3)
O2—C9—C4	114.2 (2)	C20—C19—H19	119.8
C8—C9—C4	120.8 (3)	C18—C19—H19	119.8
O2—C10—C11	112.0 (3)	C21—C20—C19	119.1 (3)
O2—C10—H10A	109.2	C21—C20—H20	120.4
C11—C10—H10A	109.2	C19—C20—H20	120.4
O2—C10—H10B	109.2	F2—C21—C20	117.7 (3)
C11—C10—H10B	109.2	F2—C21—C16	119.0 (3)
H10A—C10—H10B	107.9	C20—C21—C16	123.3 (3)
C10—C11—H11A	109.5		
			
C2—O1—C1—O3	−2.5 (5)	C6—C7—C12—C14	−0.3 (4)
C2—O1—C1—C16	175.2 (2)	C8—C7—C12—C14	−179.3 (3)
C1—O1—C2—C3	−120.5 (3)	C6—C7—C12—C15	−120.3 (3)
O1—C2—C3—N1	−58.2 (3)	C8—C7—C12—C15	60.6 (4)
O1—C2—C3—C4	177.9 (2)	C6—C7—C12—C13	120.1 (3)
N1—C3—C4—C5	−24.0 (4)	C8—C7—C12—C13	−58.9 (4)
C2—C3—C4—C5	98.7 (3)	O3—C1—C16—C17	−148.9 (4)
N1—C3—C4—C9	155.1 (2)	O1—C1—C16—C17	33.5 (4)
C2—C3—C4—C9	−82.2 (3)	O3—C1—C16—C21	33.7 (5)
C9—C4—C5—C6	−1.0 (4)	O1—C1—C16—C21	−144.0 (3)
C3—C4—C5—C6	178.1 (3)	C21—C16—C17—F1	−177.2 (3)
C4—C5—C6—C7	0.6 (5)	C1—C16—C17—F1	5.3 (4)
C5—C6—C7—C8	0.4 (4)	C21—C16—C17—C18	0.9 (5)
C5—C6—C7—C12	−178.6 (3)	C1—C16—C17—C18	−176.6 (3)
C6—C7—C8—C9	−0.9 (4)	F1—C17—C18—C19	179.1 (3)
C12—C7—C8—C9	178.1 (3)	C16—C17—C18—C19	1.0 (5)
C10—O2—C9—C8	19.4 (4)	C17—C18—C19—C20	−1.5 (5)
C10—O2—C9—C4	−161.5 (3)	C18—C19—C20—C21	0.1 (5)
C7—C8—C9—O2	179.5 (3)	C19—C20—C21—F2	179.9 (3)
C7—C8—C9—C4	0.5 (4)	C19—C20—C21—C16	2.1 (5)
C5—C4—C9—O2	−178.6 (3)	C17—C16—C21—F2	179.7 (3)
C3—C4—C9—O2	2.2 (4)	C1—C16—C21—F2	−2.7 (4)
C5—C4—C9—C8	0.5 (4)	C17—C16—C21—C20	−2.5 (4)
C3—C4—C9—C8	−178.7 (3)	C1—C16—C21—C20	175.2 (3)
C9—O2—C10—C11	67.6 (3)		

### 2-Azaniumyl-2-(4-tert-butyl-2-ethoxyphenyl)ethyl 2,6-difluorobenzoate bromide (I) Hydrogen-bond geometry (Å, º)

**Table:**

*D*—H···*A*	*D*—H	H···*A*	*D*···*A*	*D*—H···*A*
N1—H1*A*···Br1^i^	0.88	2.57	3.337 (2)	147
N1—H1*A*···F1^ii^	0.88	2.52	3.073 (3)	122
N1—H1*B*···Br1^ii^	0.88	2.39	3.259 (2)	170
N1—H1*C*···Br1	0.88	2.38	3.252 (2)	175
C10—H10*A*···Br1^iii^	0.99	2.75	3.650 (3)	152
C2—H2*B*···O2	0.99	2.62	3.116 (4)	111
C8—H8···Br1^iii^	0.95	3.10	4.040 (3)	170

Symmetry codes: (i) −*x*+1, −*y*+1, *z*; (ii) −*y*+1, *x*, −*z*+1; (iii) *x*−1/2, −*y*+3/2, −*z*+1/2.

### 2-Azaniumyl-2-(4-tert-butyl-2-ethoxyphenyl)ethyl 2,6-difluorobenzoate 3-carboxyprop-2-enoate (II) Crystal data

**Table:**

C_21_H_26_F_2_NO_3_^+^·C_4_H_3_O_4_^−^	*F*(000) = 1040
*M**_r_* = 493.49	*D*_x_ = 1.347 Mg m^−^^3^
Monoclinic, *P*2_1_/*n*	Mo *K*α radiation, λ = 0.71073 Å
*a* = 12.0615 (2) Å	Cell parameters from 9544 reflections
*b* = 15.3673 (3) Å	θ = 2.2–27.4°
*c* = 14.0141 (3) Å	µ = 0.11 mm^−^^1^
β = 110.506 (1)°	*T* = 100 K
*V* = 2432.96 (8) Å^3^	Irregular block, colourless
*Z* = 4	0.19 × 0.18 × 0.12 mm

### 2-Azaniumyl-2-(4-tert-butyl-2-ethoxyphenyl)ethyl 2,6-difluorobenzoate 3-carboxyprop-2-enoate (II) Data collection

**Table:**

Bruker D8 Venture dual source diffractometer	5576 independent reflections
Radiation source: microsource	4976 reflections with *I* > 2σ(*I*)
Detector resolution: 7.41 pixels mm^-1^	*R*_int_ = 0.031
φ and ω scans	θ_max_ = 27.5°, θ_min_ = 1.9°
Absorption correction: multi-scan (*SADABS*; Krause *et al*., 2015)	*h* = −15→14
*T*_min_ = 0.917, *T*_max_ = 0.971	*k* = −19→19
42339 measured reflections	*l* = −18→18

### 2-Azaniumyl-2-(4-tert-butyl-2-ethoxyphenyl)ethyl 2,6-difluorobenzoate 3-carboxyprop-2-enoate (II) Refinement

**Table:**

Refinement on *F*^2^	Primary atom site location: structure-invariant direct methods
Least-squares matrix: full	Secondary atom site location: difference Fourier map
*R*[*F*^2^ > 2σ(*F*^2^)] = 0.036	Hydrogen site location: difference Fourier map
*wR*(*F*^2^) = 0.101	H atoms treated by a mixture of independent and constrained refinement
*S* = 1.03	*w* = 1/[σ^2^(*F*_o_^2^) + (0.0478*P*)^2^ + 0.9503*P*] where *P* = (*F*_o_^2^ + 2*F*_c_^2^)/3
5576 reflections	(Δ/σ)_max_ = 0.001
324 parameters	Δρ_max_ = 0.34 e Å^−^^3^
0 restraints	Δρ_min_ = −0.21 e Å^−^^3^

### 2-Azaniumyl-2-(4-tert-butyl-2-ethoxyphenyl)ethyl 2,6-difluorobenzoate 3-carboxyprop-2-enoate (II) Special details

**Table:**

Experimental. The crystal was mounted using polyisobutene oil on the tip of a fine glass fibre, which was fastened in a copper mounting pin with electrical solder. It was placed directly into the cold gas stream of a liquid-nitrogen based cryostat (Hope, 1994; Parkin & Hope, 1998). Diffraction data were collected with the crystal at 100K.
Geometry. All esds (except the esd in the dihedral angle between two l.s. planes) are estimated using the full covariance matrix. The cell esds are taken into account individually in the estimation of esds in distances, angles and torsion angles; correlations between esds in cell parameters are only used when they are defined by crystal symmetry. An approximate (isotropic) treatment of cell esds is used for estimating esds involving l.s. planes.
Refinement. Refinement progress was checked using *Platon* (Spek, 2020) and by an *R*-tensor (Parkin, 2000). The final model was further checked with the IUCr utility *checkCIF*.

### 2-Azaniumyl-2-(4-tert-butyl-2-ethoxyphenyl)ethyl 2,6-difluorobenzoate 3-carboxyprop-2-enoate (II) Fractional atomic coordinates and isotropic or equivalent isotropic displacement parameters (Å^2^)

**Table:**

	*x*	*y*	*z*	*U*_iso_*/*U*_eq_	
F1	0.67039 (8)	0.57077 (6)	0.21102 (6)	0.0434 (2)	
F2	0.85345 (9)	0.79866 (6)	0.43330 (9)	0.0529 (3)	
O1	0.76401 (7)	0.55478 (5)	0.41727 (6)	0.02341 (18)	
O2	0.88984 (7)	0.39851 (5)	0.69176 (6)	0.02211 (18)	
O3	0.93824 (8)	0.62310 (6)	0.45018 (8)	0.0378 (2)	
N1	0.67234 (8)	0.48113 (6)	0.56665 (7)	0.01848 (19)	
H1A	0.6474 (7)	0.5332 (5)	0.5391 (4)	0.028*	
H1B	0.7273 (5)	0.4881 (5)	0.6284 (6)	0.028*	
H1C	0.6112 (8)	0.4514 (4)	0.5722 (5)	0.028*	
C1	0.83341 (10)	0.61834 (8)	0.40508 (9)	0.0249 (2)	
C2	0.82292 (10)	0.48524 (7)	0.48618 (9)	0.0231 (2)	
H2A	0.875467	0.509055	0.552164	0.028*	
H2B	0.870751	0.449240	0.456488	0.028*	
C3	0.72419 (10)	0.43206 (7)	0.50020 (8)	0.0193 (2)	
H3	0.660726	0.426428	0.431710	0.023*	
C4	0.75887 (9)	0.34111 (7)	0.54008 (8)	0.0185 (2)	
C5	0.70565 (10)	0.26951 (7)	0.48146 (8)	0.0221 (2)	
H5	0.645992	0.278477	0.416717	0.026*	
C6	0.73754 (10)	0.18537 (7)	0.51519 (9)	0.0230 (2)	
H6	0.699717	0.137722	0.473208	0.028*	
C7	0.82469 (10)	0.16949 (7)	0.61028 (8)	0.0198 (2)	
C8	0.87624 (10)	0.24084 (7)	0.67047 (8)	0.0195 (2)	
H8	0.934267	0.231802	0.736020	0.023*	
C9	0.84405 (9)	0.32533 (7)	0.63609 (8)	0.0185 (2)	
C10	0.99214 (10)	0.38980 (8)	0.78240 (9)	0.0252 (2)	
H10A	1.051536	0.351424	0.769867	0.030*	
H10B	0.969971	0.364617	0.838243	0.030*	
C11	1.04123 (13)	0.48013 (9)	0.80978 (12)	0.0401 (4)	
H11A	1.057683	0.505607	0.752027	0.060*	
H11B	1.114639	0.477314	0.869048	0.060*	
H11C	0.983333	0.516271	0.826106	0.060*	
C12	0.86045 (11)	0.07546 (7)	0.64402 (9)	0.0231 (2)	
C13	0.90985 (13)	0.03290 (9)	0.56848 (10)	0.0325 (3)	
H13A	0.849949	0.034849	0.499884	0.049*	
H13B	0.930531	−0.027790	0.588232	0.049*	
H13C	0.980690	0.064420	0.568981	0.049*	
C14	0.75153 (12)	0.02405 (8)	0.64565 (10)	0.0304 (3)	
H14A	0.689378	0.026624	0.578204	0.046*	
H14B	0.722055	0.049559	0.696287	0.046*	
H14C	0.773838	−0.036733	0.663484	0.046*	
C15	0.95581 (12)	0.07098 (8)	0.75090 (9)	0.0282 (3)	
H15A	0.975912	0.010015	0.769547	0.042*	
H15B	0.925608	0.097883	0.800339	0.042*	
H15C	1.026686	0.102185	0.750946	0.042*	
C16	0.76319 (11)	0.68173 (8)	0.32678 (10)	0.0262 (3)	
C17	0.68208 (12)	0.65686 (10)	0.2331 (1)	0.0333 (3)	
C18	0.61531 (13)	0.71439 (12)	0.16093 (12)	0.0452 (4)	
H18	0.560383	0.694544	0.097881	0.054*	
C19	0.63056 (15)	0.80174 (12)	0.18290 (15)	0.0521 (5)	
H19	0.583635	0.842656	0.134830	0.062*	
C20	0.71214 (15)	0.83136 (11)	0.27283 (16)	0.0509 (4)	
H20	0.723916	0.891948	0.285955	0.061*	
C21	0.77655 (13)	0.77084 (9)	0.34364 (12)	0.0375 (3)	
O4	0.47353 (7)	0.38720 (5)	0.53105 (6)	0.02278 (18)	
O5	0.40305 (8)	0.25232 (6)	0.53032 (6)	0.0292 (2)	
O6	0.74564 (8)	0.26663 (5)	0.85732 (6)	0.0274 (2)	
H6A	0.8000 (14)	0.2498 (4)	0.9192 (13)	0.041*	
O7	0.68739 (8)	0.12803 (6)	0.85259 (7)	0.0313 (2)	
C22	0.47330 (9)	0.31260 (7)	0.56934 (8)	0.0192 (2)	
C23	0.56755 (9)	0.29645 (7)	0.67107 (8)	0.0197 (2)	
H23	0.615376	0.343869	0.705630	0.024*	
C24	0.58657 (10)	0.21927 (7)	0.71429 (8)	0.0217 (2)	
H24	0.538305	0.172432	0.678666	0.026*	
C25	0.67817 (10)	0.20027 (7)	0.81480 (8)	0.0213 (2)	

### 2-Azaniumyl-2-(4-tert-butyl-2-ethoxyphenyl)ethyl 2,6-difluorobenzoate 3-carboxyprop-2-enoate (II) Atomic displacement parameters (Å^2^)

**Table:**

	*U*^11^	*U*^22^	*U*^33^	*U*^12^	*U*^13^	*U*^23^
F1	0.0488 (5)	0.0455 (5)	0.0311 (4)	−0.0028 (4)	0.0082 (4)	−0.0020 (4)
F2	0.0510 (6)	0.0265 (4)	0.0756 (7)	−0.0040 (4)	0.0153 (5)	−0.0059 (4)
O1	0.0237 (4)	0.0208 (4)	0.0242 (4)	0.0011 (3)	0.0066 (3)	0.0057 (3)
O2	0.0215 (4)	0.0164 (4)	0.0205 (4)	0.0018 (3)	−0.0026 (3)	−0.0011 (3)
O3	0.0224 (4)	0.0356 (5)	0.0520 (6)	0.0002 (4)	0.0089 (4)	0.0142 (5)
N1	0.0176 (4)	0.0158 (4)	0.0195 (4)	0.0016 (3)	0.0033 (3)	0.0016 (3)
C1	0.0246 (6)	0.0222 (6)	0.0295 (6)	0.0015 (4)	0.0116 (5)	0.0033 (5)
C2	0.0226 (5)	0.0202 (5)	0.0256 (6)	0.0035 (4)	0.0072 (4)	0.0048 (4)
C3	0.0214 (5)	0.0167 (5)	0.0173 (5)	0.0015 (4)	0.0037 (4)	0.0002 (4)
C4	0.0193 (5)	0.0166 (5)	0.0182 (5)	0.0018 (4)	0.0049 (4)	0.0011 (4)
C5	0.0244 (5)	0.0196 (5)	0.0175 (5)	0.0012 (4)	0.0015 (4)	0.0001 (4)
C6	0.0281 (6)	0.0170 (5)	0.0198 (5)	−0.0003 (4)	0.0031 (4)	−0.0019 (4)
C7	0.0216 (5)	0.0175 (5)	0.0199 (5)	0.0021 (4)	0.0069 (4)	0.0006 (4)
C8	0.0191 (5)	0.0194 (5)	0.0178 (5)	0.0025 (4)	0.0038 (4)	0.0012 (4)
C9	0.0179 (5)	0.0171 (5)	0.0191 (5)	0.0002 (4)	0.0049 (4)	−0.0015 (4)
C10	0.0219 (5)	0.0206 (5)	0.0233 (5)	0.0013 (4)	−0.0043 (4)	0.0003 (4)
C11	0.0325 (7)	0.0236 (6)	0.0436 (8)	−0.0011 (5)	−0.0123 (6)	0.0007 (6)
C12	0.0288 (6)	0.0166 (5)	0.0215 (5)	0.0038 (4)	0.0057 (4)	0.0006 (4)
C13	0.0413 (7)	0.0276 (6)	0.0283 (6)	0.0116 (5)	0.0119 (5)	0.0006 (5)
C14	0.0354 (7)	0.0228 (6)	0.0299 (6)	−0.0009 (5)	0.0078 (5)	0.0039 (5)
C15	0.0341 (6)	0.0212 (6)	0.0248 (6)	0.0056 (5)	0.0047 (5)	0.0040 (5)
C16	0.0238 (6)	0.0247 (6)	0.0339 (6)	0.0033 (5)	0.0151 (5)	0.0092 (5)
C17	0.0302 (6)	0.0433 (8)	0.0305 (6)	0.0035 (6)	0.0155 (5)	0.0113 (6)
C18	0.0337 (7)	0.0687 (11)	0.0364 (8)	0.0105 (7)	0.0162 (6)	0.0246 (7)
C19	0.0404 (8)	0.0587 (11)	0.0656 (11)	0.0195 (8)	0.0292 (8)	0.0401 (9)
C20	0.0474 (9)	0.0308 (8)	0.0837 (13)	0.0131 (7)	0.0342 (9)	0.0248 (8)
C21	0.0330 (7)	0.0286 (7)	0.0536 (9)	0.0012 (5)	0.0188 (6)	0.0072 (6)
O4	0.0183 (4)	0.0208 (4)	0.0251 (4)	0.0018 (3)	0.0023 (3)	0.0076 (3)
O5	0.0288 (4)	0.0240 (4)	0.0226 (4)	−0.0066 (3)	−0.0063 (3)	0.0042 (3)
O6	0.0294 (4)	0.0214 (4)	0.0199 (4)	−0.0031 (3)	−0.0060 (3)	0.0028 (3)
O7	0.0328 (5)	0.0211 (4)	0.0286 (4)	−0.0010 (4)	−0.0035 (4)	0.0078 (3)
C22	0.0179 (5)	0.0191 (5)	0.0181 (5)	0.0016 (4)	0.0030 (4)	0.0024 (4)
C23	0.0187 (5)	0.0190 (5)	0.0174 (5)	−0.0016 (4)	0.0014 (4)	0.0000 (4)
C24	0.0215 (5)	0.0192 (5)	0.0187 (5)	−0.0021 (4)	0.0002 (4)	0.0007 (4)
C25	0.0216 (5)	0.0203 (5)	0.0188 (5)	0.0006 (4)	0.0032 (4)	0.0017 (4)

### 2-Azaniumyl-2-(4-tert-butyl-2-ethoxyphenyl)ethyl 2,6-difluorobenzoate 3-carboxyprop-2-enoate (II) Geometric parameters (Å, º)

**Table:**

F1—C17	1.3547 (17)	C11—H11C	0.9800
F2—C21	1.3436 (19)	C12—C13	1.5314 (17)
O1—C1	1.3357 (14)	C12—C15	1.5376 (16)
O1—C2	1.4488 (13)	C12—C14	1.5400 (17)
O2—C9	1.3698 (13)	C13—H13A	0.9800
O2—C10	1.4338 (13)	C13—H13B	0.9800
O3—C1	1.2015 (15)	C13—H13C	0.9800
N1—C3	1.4950 (14)	C14—H14A	0.9800
N1—H1A	0.893 (8)	C14—H14B	0.9800
N1—H1B	0.893 (8)	C14—H14C	0.9800
N1—H1C	0.893 (8)	C15—H15A	0.9800
C1—C16	1.4898 (16)	C15—H15B	0.9800
C2—C3	1.5127 (16)	C15—H15C	0.9800
C2—H2A	0.9900	C16—C17	1.3881 (19)
C2—H2B	0.9900	C16—C21	1.3894 (19)
C3—C4	1.5089 (15)	C17—C18	1.372 (2)
C3—H3	1.0000	C18—C19	1.375 (3)
C4—C5	1.3886 (15)	C18—H18	0.9500
C4—C9	1.3986 (15)	C19—C20	1.377 (3)
C5—C6	1.3841 (16)	C19—H19	0.9500
C5—H5	0.9500	C20—C21	1.383 (2)
C6—C7	1.3996 (15)	C20—H20	0.9500
C6—H6	0.9500	O4—C22	1.2661 (13)
C7—C8	1.3903 (15)	O5—C22	1.2452 (14)
C7—C12	1.5345 (15)	O6—C25	1.3106 (14)
C8—C9	1.3920 (15)	O6—H6A	0.922 (18)
C8—H8	0.9500	O7—C25	1.2182 (14)
C10—C11	1.5052 (17)	C22—C23	1.5003 (14)
C10—H10A	0.9900	C23—C24	1.3149 (16)
C10—H10B	0.9900	C23—H23	0.9500
C11—H11A	0.9800	C24—C25	1.4831 (15)
C11—H11B	0.9800	C24—H24	0.9500
			
C1—O1—C2	116.21 (9)	C13—C12—C15	108.35 (10)
C9—O2—C10	118.56 (8)	C7—C12—C15	112.08 (9)
C3—N1—H1A	109.5	C13—C12—C14	109.37 (10)
C3—N1—H1B	109.5	C7—C12—C14	109.78 (10)
H1A—N1—H1B	109.5	C15—C12—C14	108.27 (10)
C3—N1—H1C	109.5	C12—C13—H13A	109.5
H1A—N1—H1C	109.5	C12—C13—H13B	109.5
H1B—N1—H1C	109.5	H13A—C13—H13B	109.5
O3—C1—O1	124.64 (11)	C12—C13—H13C	109.5
O3—C1—C16	124.72 (11)	H13A—C13—H13C	109.5
O1—C1—C16	110.63 (10)	H13B—C13—H13C	109.5
O1—C2—C3	105.09 (9)	C12—C14—H14A	109.5
O1—C2—H2A	110.7	C12—C14—H14B	109.5
C3—C2—H2A	110.7	H14A—C14—H14B	109.5
O1—C2—H2B	110.7	C12—C14—H14C	109.5
C3—C2—H2B	110.7	H14A—C14—H14C	109.5
H2A—C2—H2B	108.8	H14B—C14—H14C	109.5
N1—C3—C4	111.02 (9)	C12—C15—H15A	109.5
N1—C3—C2	109.19 (9)	C12—C15—H15B	109.5
C4—C3—C2	114.82 (9)	H15A—C15—H15B	109.5
N1—C3—H3	107.2	C12—C15—H15C	109.5
C4—C3—H3	107.2	H15A—C15—H15C	109.5
C2—C3—H3	107.2	H15B—C15—H15C	109.5
C5—C4—C9	117.6 (1)	C17—C16—C21	115.70 (12)
C5—C4—C3	120.28 (9)	C17—C16—C1	123.17 (12)
C9—C4—C3	122.13 (10)	C21—C16—C1	121.13 (12)
C6—C5—C4	121.51 (10)	F1—C17—C18	118.27 (14)
C6—C5—H5	119.2	F1—C17—C16	117.86 (12)
C4—C5—H5	119.2	C18—C17—C16	123.85 (15)
C5—C6—C7	120.95 (10)	C17—C18—C19	117.77 (16)
C5—C6—H6	119.5	C17—C18—H18	121.1
C7—C6—H6	119.5	C19—C18—H18	121.1
C8—C7—C6	117.86 (10)	C18—C19—C20	121.64 (14)
C8—C7—C12	122.55 (10)	C18—C19—H19	119.2
C6—C7—C12	119.6 (1)	C20—C19—H19	119.2
C7—C8—C9	120.95 (10)	C19—C20—C21	118.43 (16)
C7—C8—H8	119.5	C19—C20—H20	120.8
C9—C8—H8	119.5	C21—C20—H20	120.8
O2—C9—C8	124.1 (1)	F2—C21—C20	119.16 (14)
O2—C9—C4	114.79 (9)	F2—C21—C16	118.27 (13)
C8—C9—C4	121.1 (1)	C20—C21—C16	122.56 (16)
O2—C10—C11	106.22 (9)	C25—O6—H6A	109.5
O2—C10—H10A	110.5	O5—C22—O4	126.13 (10)
C11—C10—H10A	110.5	O5—C22—C23	117.83 (10)
O2—C10—H10B	110.5	O4—C22—C23	116.04 (10)
C11—C10—H10B	110.5	C24—C23—C22	122.6 (1)
H10A—C10—H10B	108.7	C24—C23—H23	118.7
C10—C11—H11A	109.5	C22—C23—H23	118.7
C10—C11—H11B	109.5	C23—C24—C25	124.52 (10)
H11A—C11—H11B	109.5	C23—C24—H24	117.7
C10—C11—H11C	109.5	C25—C24—H24	117.7
H11A—C11—H11C	109.5	O7—C25—O6	124.32 (10)
H11B—C11—H11C	109.5	O7—C25—C24	121.09 (10)
C13—C12—C7	108.94 (10)	O6—C25—C24	114.58 (10)
			
C2—O1—C1—O3	−3.44 (18)	C8—C7—C12—C15	1.27 (16)
C2—O1—C1—C16	175.71 (10)	C6—C7—C12—C15	−179.17 (11)
C1—O1—C2—C3	170.25 (9)	C8—C7—C12—C14	121.63 (12)
O1—C2—C3—N1	−73.64 (10)	C6—C7—C12—C14	−58.81 (14)
O1—C2—C3—C4	160.96 (9)	O3—C1—C16—C17	132.61 (14)
N1—C3—C4—C5	118.11 (11)	O1—C1—C16—C17	−46.55 (16)
C2—C3—C4—C5	−117.45 (12)	O3—C1—C16—C21	−46.75 (19)
N1—C3—C4—C9	−61.61 (13)	O1—C1—C16—C21	134.09 (12)
C2—C3—C4—C9	62.83 (14)	C21—C16—C17—F1	176.68 (11)
C9—C4—C5—C6	−1.68 (17)	C1—C16—C17—F1	−2.72 (18)
C3—C4—C5—C6	178.60 (11)	C21—C16—C17—C18	−1.76 (19)
C4—C5—C6—C7	0.26 (18)	C1—C16—C17—C18	178.84 (12)
C5—C6—C7—C8	1.34 (17)	F1—C17—C18—C19	−178.03 (12)
C5—C6—C7—C12	−178.24 (11)	C16—C17—C18—C19	0.4 (2)
C6—C7—C8—C9	−1.49 (16)	C17—C18—C19—C20	1.8 (2)
C12—C7—C8—C9	178.08 (10)	C18—C19—C20—C21	−2.4 (2)
C10—O2—C9—C8	12.17 (16)	C19—C20—C21—F2	−177.63 (14)
C10—O2—C9—C4	−168.79 (10)	C19—C20—C21—C16	0.9 (2)
C7—C8—C9—O2	179.04 (10)	C17—C16—C21—F2	179.64 (12)
C7—C8—C9—C4	0.06 (17)	C1—C16—C21—F2	−0.95 (19)
C5—C4—C9—O2	−177.55 (10)	C17—C16—C21—C20	1.1 (2)
C3—C4—C9—O2	2.17 (15)	C1—C16—C21—C20	−179.53 (13)
C5—C4—C9—C8	1.52 (16)	O5—C22—C23—C24	7.78 (17)
C3—C4—C9—C8	−178.76 (10)	O4—C22—C23—C24	−171.94 (11)
C9—O2—C10—C11	164.55 (11)	C22—C23—C24—C25	−179.7 (1)
C8—C7—C12—C13	−118.63 (12)	C23—C24—C25—O7	176.53 (12)
C6—C7—C12—C13	60.93 (14)	C23—C24—C25—O6	−3.43 (17)

### 2-Azaniumyl-2-(4-tert-butyl-2-ethoxyphenyl)ethyl 2,6-difluorobenzoate 3-carboxyprop-2-enoate (II) Hydrogen-bond geometry (Å, º)

**Table:**

*D*—H···*A*	*D*—H	H···*A*	*D*···*A*	*D*—H···*A*
N1—H1*A*···O4^i^	0.89	1.89	2.7160 (12)	152
N1—H1*B*···O2	0.89	2.30	2.8889 (12)	123
N1—H1*B*···O7^ii^	0.89	2.36	2.8103 (13)	112
N1—H1*C*···O4	0.89	1.84	2.6915 (12)	158
C2—H2*A*···O7^ii^	0.99	2.53	3.1832 (15)	123
C2—H2*B*···O3^iii^	0.99	2.48	3.1727 (15)	127
O6—H6*A*···O5^iv^	0.92	1.62	2.5184 (11)	165

Symmetry codes: (i) −*x*+1, −*y*+1, −*z*+1; (ii) −*x*+3/2, *y*+1/2, −*z*+3/2; (iii) −*x*+2, −*y*+1, −*z*+1; (iv) *x*+1/2, −*y*+1/2, *z*+1/2.
